# Differences in Rheological Properties between Brand and Generic Metronidazole Gels and Their Potential Impact on Malodor Management in Cancer Survivors

**DOI:** 10.31662/jmaj.2025-0563

**Published:** 2026-05-22

**Authors:** Atsushi Ishimura, Shiori Obuchi, Naohiro Yabuki, Motoki Inoue

**Affiliations:** 1 Laboratory of Clinical Pharmacy Assessment, Hoshi University, Shinagawa-Ku, Tokyo, Japan; 2 Pharmaceutical Department of Sainokuni Higashi Omiya Medical Center, Saitama-shi, Saitama, Japan; 3 Department of Physical Chemistry, Hoshi University, Shinagawa-Ku, Tokyo, Japan

**Keywords:** topical semisolid formulation, metronidazole gel, rheology / Q3 characterization, gel network structure, polymer excipients

## Abstract

**Introduction:**

Malignant fungating wounds often produce strong malodors caused by anaerobic bacterial proliferation, which severely impairs the quality of life of cancer survivors and their caregivers. Topical metronidazole is widely used as a first-line option for odor control; however, differences in the rheological (Q3) attributes between brand-name and generic gel formulations may influence spreadability, retention on the wound surface, and overall usability. The study aimed to compare the rheological properties of brand and generic metronidazole gels marketed in Japan and to discuss their potential impact on malodor management.

**Methods:**

Brand (Rozex^Ⓡ^ Gel 0.75%) and generic metronidazole gels were evaluated using a rotational rheometer. Flow behavior, thixotropy, viscoelasticity (G′, G″), linear viscoelastic region (LVR), and frequency sweep responses were measured at 25℃. Excipients were compared qualitatively based on package inserts.

**Results:**

Both formulations exhibited shear-thinning behavior; however, the brand gel showed consistently higher viscosity across all shear rates, with a pronounced difference at low shear rates relevant to wound application. The brand product demonstrated a narrower thixotropic hysteresis loop (approximately 26-fold difference), indicating faster structural recovery. Viscoelastic measurements showed higher G′ values and a wider LVR for the brand gel, suggesting a stronger gel network. Frequency sweep analysis revealed strong-gel behavior for the brand formulation, whereas the generic gel exhibited weak-gel characteristics. These rheological differences corresponded to differences in base polymers―carboxyvinyl polymer in the brand gel and hydrophobically modified hydroxypropyl methylcellulose in the generic.

**Conclusions:**

Although containing identical active ingredients, the two formulations exhibit distinct rheological profiles that may affect clinical handling and usability in malodor management. Q3 attributes should be considered when selecting topical products for cancer survivors with fungating wounds.

## Introduction

Malignant fungating wounds arise when cancer cells infiltrate the skin directly or develop as cutaneous metastases, affecting approximately 5%-14% of patients with advanced malignancies ^(1), (2)^. These wounds, particularly those associated with advanced breast cancer or chest wall recurrence, often present with ulceration, bleeding, necrosis, infection, excessive exudate, and a characteristic strong malodor ^(3), (4)^. Among these distressing symptoms, malodor is frequently reported as the most debilitating. It compromises not only daily functioning for patients but also imposes significant psychological burdens on caregivers, contributing to embarrassment, social withdrawal, anxiety, and sleep disturbances ^(5), (6)^. The odor primarily results from anaerobic bacterial proliferation within necrotic tissue, where volatile amines such as putrescine and cadaverine are generated ^(7)^. Effective odor control is therefore essential in supportive care for cancer survivors with fungating wounds ^(8)^.

Topical metronidazole has long been used as a first-line therapy for odor management due to its potent activity against anaerobic bacteria ^(9), (10)^. Its antibacterial effects are mediated by reductive activation of the nitro group, leading to inhibition of deoxyribonucleic acid synthesis and the formation of cytotoxic radicals ^(11)^. In Japan, topical metronidazole products were unavailable for many years, and hospital-compounded preparations were commonly used. The release of the brand-name metronidazole gel (Rozex^Ⓡ^ Gel) in 2015, followed by the introduction of generic formulations in 2025, has provided standardized options for clinical practice. Post-marketing surveillance has demonstrated favorable safety, with adverse event rates of approximately 3%, and high levels of therapeutic effectiveness and patient satisfaction exceeding 70% ^(12)^. Thus, topical metronidazole is now well established as a safe and effective supportive care intervention.

Unlike systemic drugs, however, topical semisolid formulations cannot be evaluated using conventional bioequivalence studies based on systemic pharmacokinetics, owing to their minimal percutaneous absorption. Regulatory assessments for generic topical products therefore rely on Q1 (qualitative composition), Q2 (quantitative composition), and Q3 (microstructural and physicochemical) equivalence, complemented by in vitro release testing and in vitro permeation testing ^(13), (14), (15)^. Q3 attributes―such as viscosity, viscoelasticity, and structural recovery―are particularly relevant because they reflect differences in bases and excipients that directly influence spreadability, retention on the wound surface, and the user’s tactile experience. Supporting this, we previously reported that variations in Q3 properties between brand-name and generic heparinoid ointments affected patient perception and usability ^(16)^.

Although metronidazole gels share the same active ingredient, differences in excipient composition and polymer structure may result in divergent rheological behaviors. These variations could, in turn, influence wound coverage, residence time, dressing stability, and ultimately the patient’s experience of malodor management. To date, however, no systematic comparison of the Q3 attributes of brand-name and generic metronidazole gels available in Japan has been conducted. The present study aims to characterize and compare the rheological properties of brand and newly marketed generic metronidazole gels, focusing on viscosity, viscoelastic moduli (G′ and G″), and structural recovery. Based on these findings, we discuss how differences in Q3 attributes may affect usability in clinical practice and potentially impact the quality of life of patients living with malignant fungating wounds.

## Materials and Methods

### Materials

Two topical metronidazole gel formulations commercially available in Japan were evaluated: the brand-name product (lot no. 5542009; Maruho Co., Ltd.) and a generic formulation (lot no. 520421; Maruishi Pharmaceutical Co., Ltd.). Both products were purchased from the market, stored at room temperature in a dark environment after opening, and analyzed within 1 month. All samples were used within their labeled expiration dates.

The qualitative composition of each gel was compared based on information provided in the official package inserts, and the excipients included in each formulation are summarized in Table 1. Quantitative ratios of excipients were not disclosed and thus were not evaluated.

**Table 1.** Excipients Contained in Each Formulation.

	Brand	Generic
Sodium edetate hydrate	〇	〇
Carboxyvinyl polymer	〇	-
Hydrophobically modified hydroxypropyl methylcellulose	-	〇
Propylene glycol	〇	〇
Methyl parahydroxybenzoate	〇	〇
Propyl parahydroxybenzoate	〇	〇
Sodium hydroxide	〇	-

〇: included; -: not included.

### Rheological measurements

Rheological characterization was conducted using a rotational rheometer (MCR302e, Anton Paar, Graz, Austria) equipped with a 25-mm parallel-plate geometry. The measurement gap was set to 1 mm. Prior to measurement, samples were briefly subjected to ultrasonication to remove entrapped air bubbles. After loading onto the lower plate, excess material was carefully trimmed, and the normal force was monitored to ensure stabilization below 1 N before initiating measurements. The loaded samples were allowed to rest for 5 minutes at 25℃ to achieve thermal equilibration and structural stabilization. All measurements were performed at a controlled temperature of 25℃. An evaporation cover supplied with the rheometer was used throughout the experiments to minimize solvent evaporation.

### Flow behavior analysis

Flow behavior was assessed by measuring viscosity while increasing and subsequently decreasing the shear rate from 1 to 10 s^-1^ in a stepwise manner. Ascending and descending curves were obtained to characterize shear-thinning behavior and to evaluate thixotropy based on the area of the hysteresis loop, which reflects structural breakdown and subsequent recovery.

### Oscillatory strain sweep

Viscoelastic properties were evaluated by applying increasing strain from 0.01% to 100% at a constant angular frequency of 1 Hz. Storage modulus (G′) and loss modulus (G″) were recorded, and the linear viscoelastic region (LVR) was identified as the range over which G′ remained essentially constant. Yield strain was defined as the strain at which G′ begins to deviate from the LVR.

### Frequency sweep analysis

Frequency-dependent viscoelasticity was assessed within the LVR by varying the angular frequency from 100 to 0.1 rad/s. The frequency responses of G′ and G″ were analyzed to evaluate the strength and stability of the gel-network structures.

## Results

### Flow behavior of brand and generic topical gels

**Figure 1.**
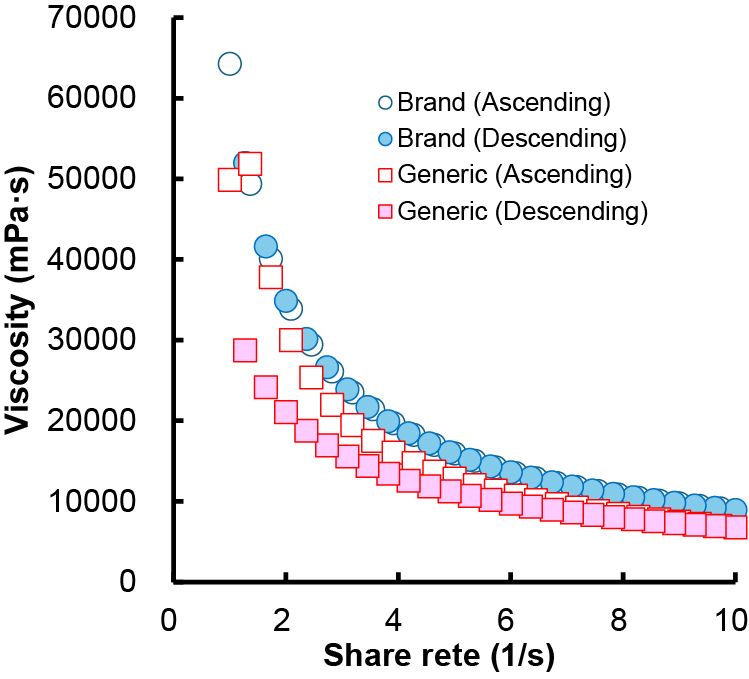
Flow behavior of Brand and Generic topical gels.

As shown in Figure 1, both the brand and generic gels exhibited typical shear-thinning behavior, with viscosity decreasing as shear rate increased. This non-Newtonian profile is characteristic of semisolid topical formulations. Across the entire shear-rate range, however, the brand gel consistently demonstrated higher viscosity than the generic formulation, with the most pronounced differences observed at low shear rates (approximately 1-3 s^-1^), which are relevant to wound application. Both formulations displayed hysteresis loops between ascending and descending curves, indicating mild thixotropy. Notably, the brand gel exhibited a smaller hysteresis area, suggesting faster viscosity recovery after structural breakdown. Thixotropy was quantified as the area enclosed by the hysteresis loop between the ascending and descending flow curves. The hysteresis loop area was 1.10 × 10^3^ for the brand gel and 2.90 × 10^4^ for the generic gel, indicating approximately a 26-fold greater thixotropy in the generic formulation, which may influence spreading behavior and structural recovery during topical application.

### Viscoelastic properties

**Figure 2.**
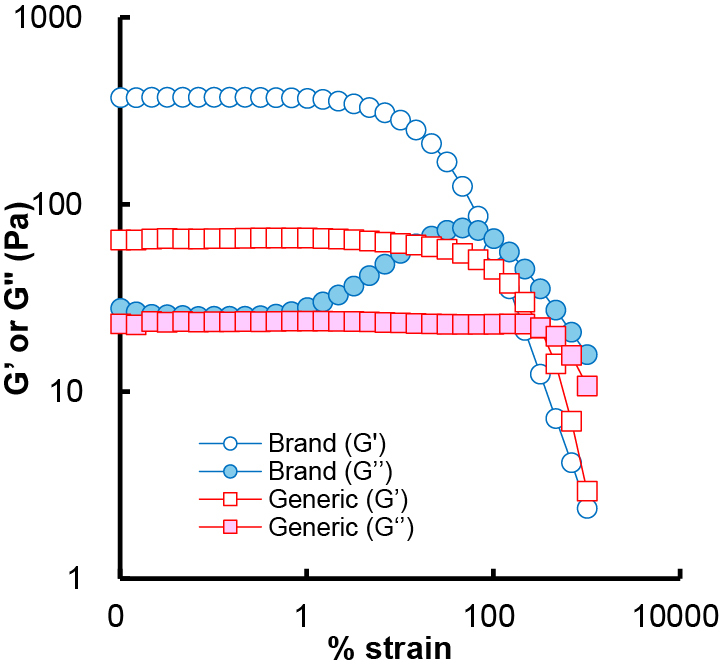
Strain sweep analysis of Brand and Generic topical gels.

Strain-sweep measurements (Figure 2) showed that, for both formulations, the storage modulus (G′) exceeded the loss modulus (G″), indicating predominantly elastic gel behavior. The brand gel displayed higher absolute G′ values, reflecting a stronger and more robust network structure. In addition, the LVR was wider for the brand formulation, and the strain at which G′ began to decrease sharply (the breakdown point) occurred at higher strain levels compared with the generic gel. These findings suggest that the brand gel better maintains its structure under external stress, whereas the generic formulation is more susceptible to structural collapse at lower strain. Yield strain, defined as the point at which G′ begins to deviate from linearity, was higher in the brand formulation, indicating greater resistance to structural deformation.

### Frequency sweep analysis

**Figure 3.**
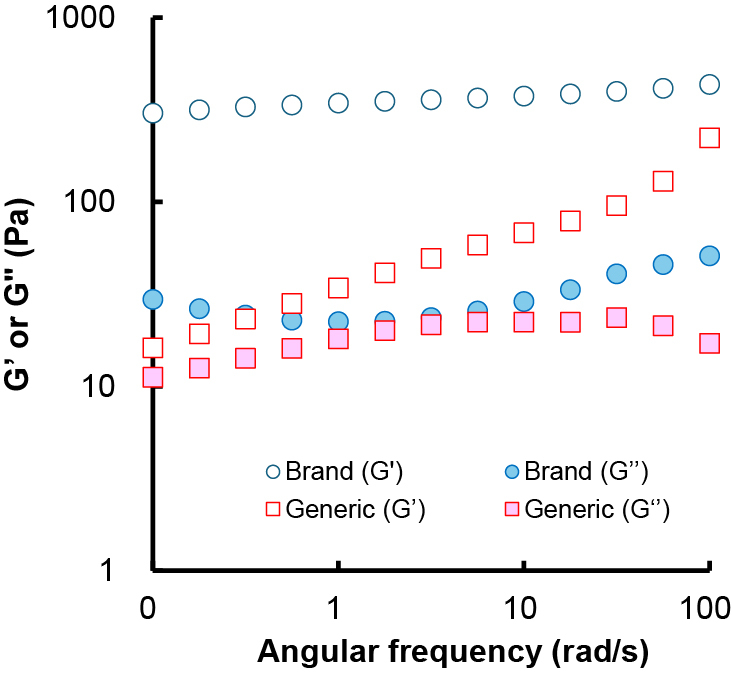
Frequency sweep profiles of Brand and Generic topical gels.

Figure 3 illustrates the frequency dependence of viscoelastic moduli. The brand gel maintained nearly constant G′ values across the tested frequency range, demonstrating minimal frequency dependence consistent with “strong-gel” behavior. In contrast, the generic gel exhibited an increase in G′ at higher frequencies and greater sensitivity to frequency changes, characteristic of a “weak-gel” network. Overall, the brand formulation possessed a more stable gel structure, whereas the generic gel showed greater structural variability. The frequency dependence of G′ and G″ further supports the stronger gel-network structure of the brand formulation compared with the more deformable network of the generic gel.

### Comparison of base ingredient composition

A qualitative comparison of excipients based on package inserts is summarized in Table 1. The brand formulation contains carboxyvinyl polymer, whereas the generic gel contains hydrophobically modified hydroxypropyl methylcellulose as the primary gelling agent. Because quantitative proportions are not disclosed, the comparison is limited to qualitative differences in polymer type.

## Discussion

### Clinical implications of rheological differences

Malodor associated with malignant fungating wounds remains a persistent challenge in supportive oncology care, affecting not only patients but also their caregivers through its impact on daily activities and psychosocial well-being ^(17), (18)^. Because topical metronidazole is widely used as a first-line approach to odor control, understanding how formulation characteristics influence real-world applicability is of practical relevance.

The present study identified consistent differences in rheological behavior between brand-name and generic metronidazole gels. The higher viscosity observed in the brand formulation at low shear rates―conditions that approximate initial application to the wound surface―suggests greater stability in areas where mechanical stress is minimal ^(19), (20)^. This may help retain the gel within regions of necrosis or exudate, which could be advantageous for maintaining its position under dressings. In contrast, the lower viscosity of the generic formulation may facilitate smoother spreading across larger or uneven wound areas. Such differences indicate that neither formulation is universally superior; rather, each may present practical advantages depending on wound characteristics, exudate volume, and user preferences.

The smaller hysteresis loop observed in the brand gel reflects more rapid recovery of structure following shear-induced disruption ^(21)^. This quantitative difference (approximately 26-fold) further supports the distinct structural recovery characteristics between the formulations. A formulation capable of regaining its consistency shortly after manipulation is less likely to run or shift after application, which may help preserve dressing integrity and reduce the need for repeated adjustments. Meanwhile, the more deformable structure of the generic gel may allow gentler application, potentially reducing discomfort in patients with sensitive or fragile wound beds.

Oscillatory measurements further demonstrated differences in gel-network robustness. The brand formulation exhibited higher storage modulus (G′) values and a wider LVR, indicating a more resilient network capable of maintaining structure under varying degrees of stress encountered during routine wound care, such as cleansing or dressing changes. By contrast, the earlier structural breakdown of the generic gel suggests a network more responsive to deformation, which may be appropriate in situations requiring minimal mechanical resistance, such as care for painful or fragile wounds.

### Influence of polymer composition on gel-network structure

The polymeric bases used in each product likely account for many of these rheological distinctions. Carboxyvinyl polymer, used in the brand formulation, typically forms a cohesive and elastic gel network, whereas hydrophobically modified Hypromellose creates structures that are more flexible but less resistant to deformation ^(22), (23)^. These findings highlight how excipient selection influences Q3 attributes and, consequently, the practical handling of topical semisolid formulations used in oncology care.

### Study limitations and future perspectives

Although these observations help contextualize how formulation-dependent properties may affect usability, several factors should temper the interpretation of the findings. Quantitative excipient ratios (Q2) were not disclosed, limiting the ability to attribute rheological behavior to specific component concentrations. Additionally, rheological performance, while informative, does not directly predict clinical outcomes such as odor reduction, wound comfort, or caregiver burden, all of which may be influenced by wound morphology, exudate level, dressing selection, and patient mobility. The absence of sensory evaluations or patient- and caregiver-reported usability assessments also restricts conclusions regarding how these rheological differences translate to everyday practice. Further work integrating rheological profiles with clinical odor assessments, user experience measures, and evaluations across variable wound conditions will help clarify the extent to which formulation attributes influence real-world outcomes in malodor management. Determination of fracture stress and other mechanical parameters would provide further insight into gel failure behavior and warrants future investigation.

## Conclusions

The brand-name and generic metronidazole gels demonstrated distinct rheological characteristics despite containing the same active ingredient. The brand formulation exhibited higher viscosity, faster structural recovery, and a stronger gel network, while the generic formulation behaved as a weaker gel with earlier structural breakdown. These differences may influence clinical handling, wound retention, and usability in malodor management. Future studies should examine associations between rheological properties, odor-control performance, and patient-reported outcomes.

## Article Information

### Author Contributions

Conceptualization: Atsushi Ishimura, Naohiro Yabuki, and Motoki Inoue. Methodology: Atsushi Ishimura and Shiori Obuchi. Validation: Motoki Inoue. Formal analysis: Motoki Inoue. Investigation: Atsushi Ishimura, Shiori Obuchi, Naohiro Yabuki, and Motoki Inoue. Resources, Motoki Inoue. Data curation: Atsushi Ishimura. Writing―original draft preparation: Atsushi Ishimura. Writing―review and editing: Motoki Inoue. Visualization: Atsushi Ishimura. Supervision: Motoki Inoue. All authors have read and agreed to the published version of the manuscript.

### Conflicts of Interest

None

### IRB Approval

IRB approval was not required as this study did not involve human subjects or identifiable personal data.

### Informed Consent

Informed consent was not required as this study did not involve human participants.

### Data Sharing Statement

The data generated and/or analyzed during the current study are not publicly available.
